# Supplementary material for: SanA is an inner membrane protein mediating Salmonella Typhimurium infection
Source: Microbiol Spectr. 2025 Apr 30;13(6):e02833-24. doi: 10.1128/spectrum.02833-24 (PMC12131732; doi:10.1128/spectrum.02833-24)
Supplement: Supplemental figure legends — All Supplementary Figures with captions. [file spectrum.02833-24-s0001.docx]

**Figure S1** Growth curves of *S*. Typhimurium 4/74 WT and *sanA*_RBS_*::luc* in LB medium. Nc indicates medium without bacteria. The data are shown as mean values and SEM of at least three separate experiments.

**Figure S2** Densitometric analysis of protein bands imaged with the ChemiDoc MP. The average relative density of SicA was compared to the relative difference in GFP quantity of protein load for the *S*. Typhimurium lysates. The data are shown as mean values and SEM of at least three separate experiments. Statistical differences were analyzed by Student’s t test (*, p<0.05)

**
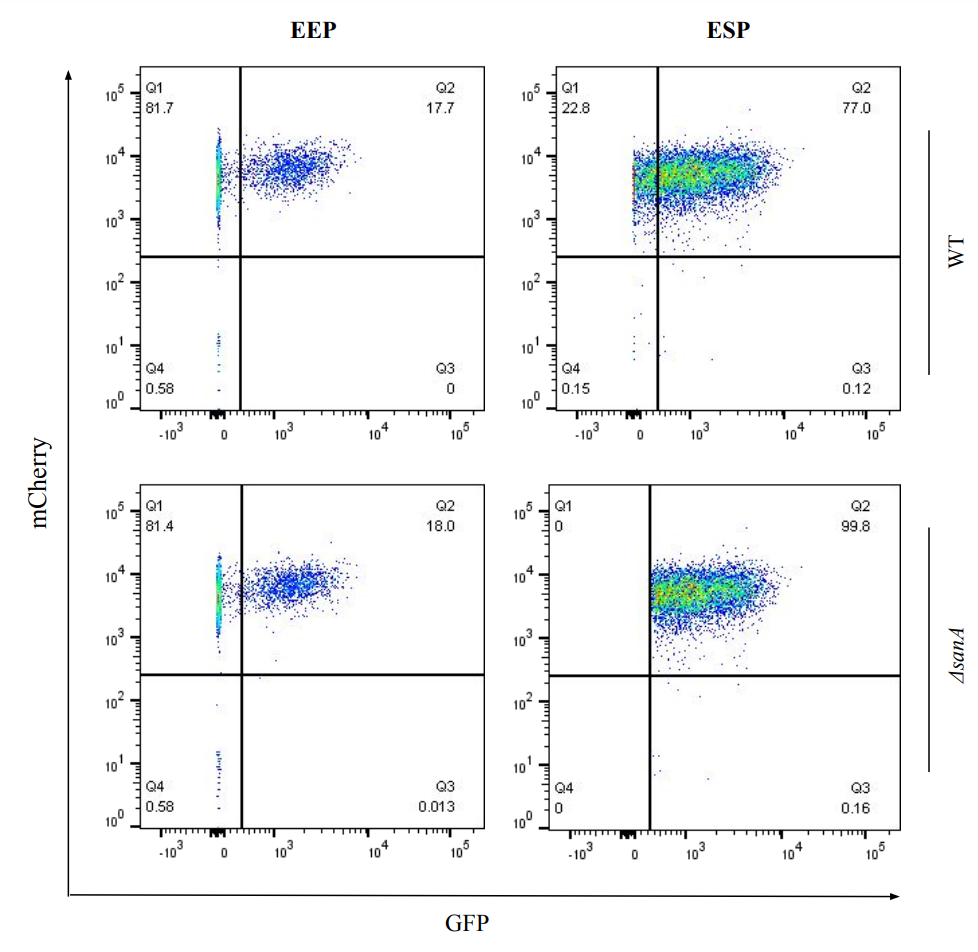
**

**Figure S3** Fraction of cells expressing *sicA*. Fraction of cells in the ON state was determined relative to the negative control (100% in the OFF state), which consisted of the measured fluorescence of cells not expressing the GFP. EEP: early exponential growth phase corresponding to OD_600_=0.5; ESP: early stationary growth phase corresponding to OD_600_=2.0.


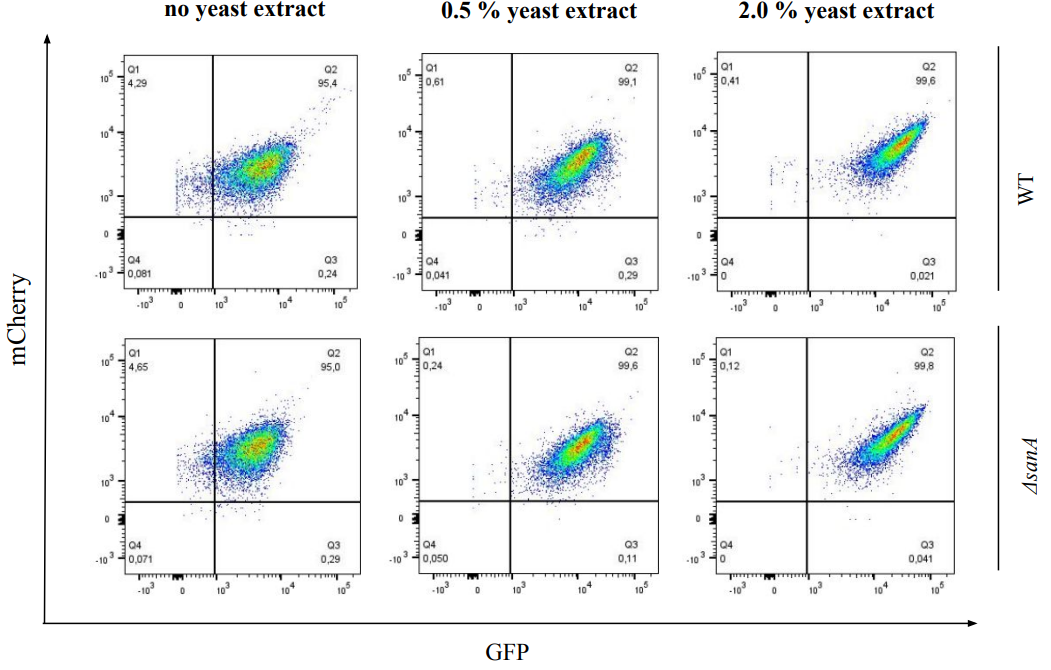


**Figure S4** Fraction of cells expressing *sicA*. Fraction of cells in the ON state was determined relative to the negative control (100% in the OFF state), which consisted of the measured fluorescence of cells not expressing the GFP.


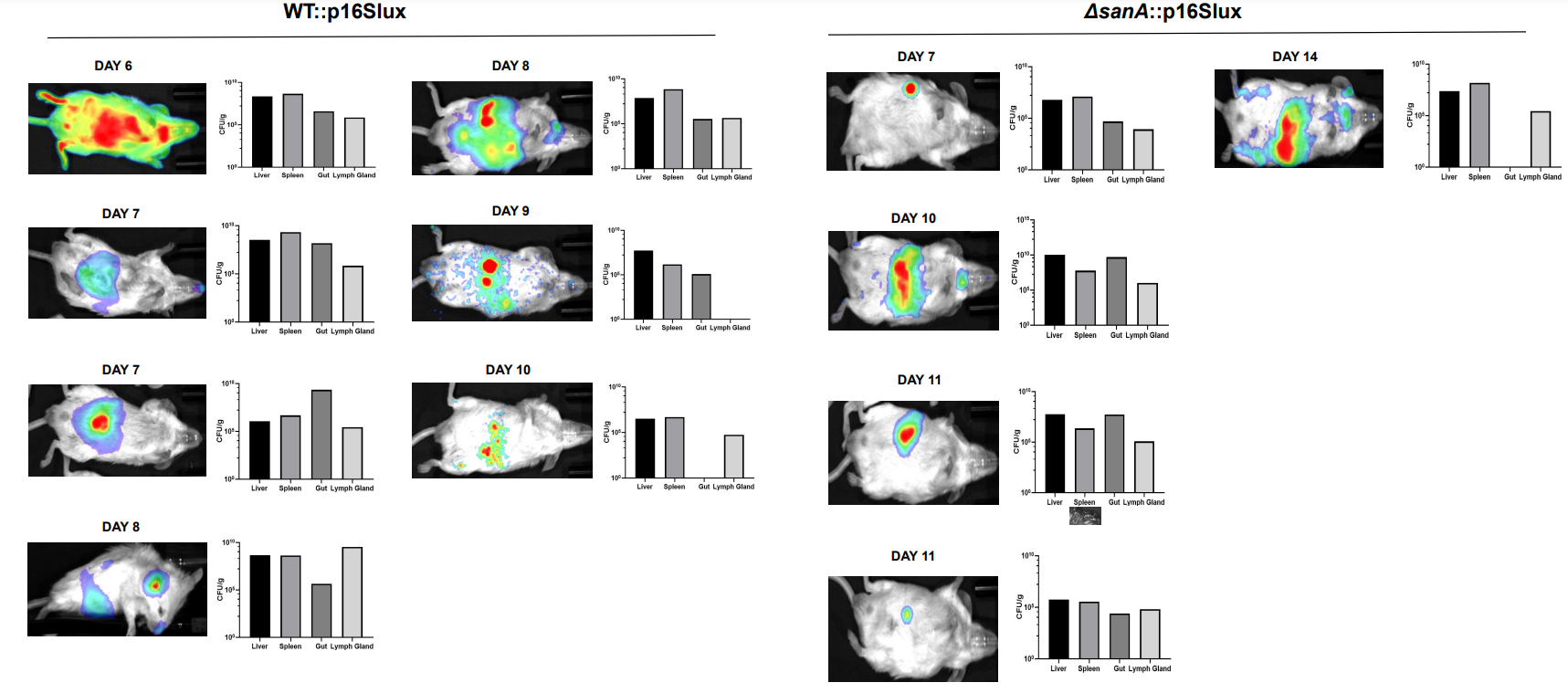


**Figure S5** Monitoring of WT::p16Slux *S*. Typhimurium and *ΔsanA*::p16Slux infections in mice using whole-body bioluminescence imaging (BLI). BALB/c mice (7 per group) were inoculated with 10^7^ CFU of *Salmonella*. Bioluminescence signals were monitored in the animals every morning for 21 days post-inoculation. Upon detection of bioluminescence, the mouse was sacrificed, and the liver, spleen, small intestine, and lymph nodes were homogenized and diluted to evaluate the bacterial load (CFU/g). The intensity of bioluminescence emission is represented as a pseudocolor image, with red indicating the highest intensity and blue the lowest.
