# Supplementary figures and images for: SanA is an inner membrane protein mediating Salmonella Typhimurium infection
Source: Microbiol Spectr. 2025 Apr 30;13(6):e02833-24. doi: 10.1128/spectrum.02833-24 (PMC12131732; doi:10.1128/spectrum.02833-24)

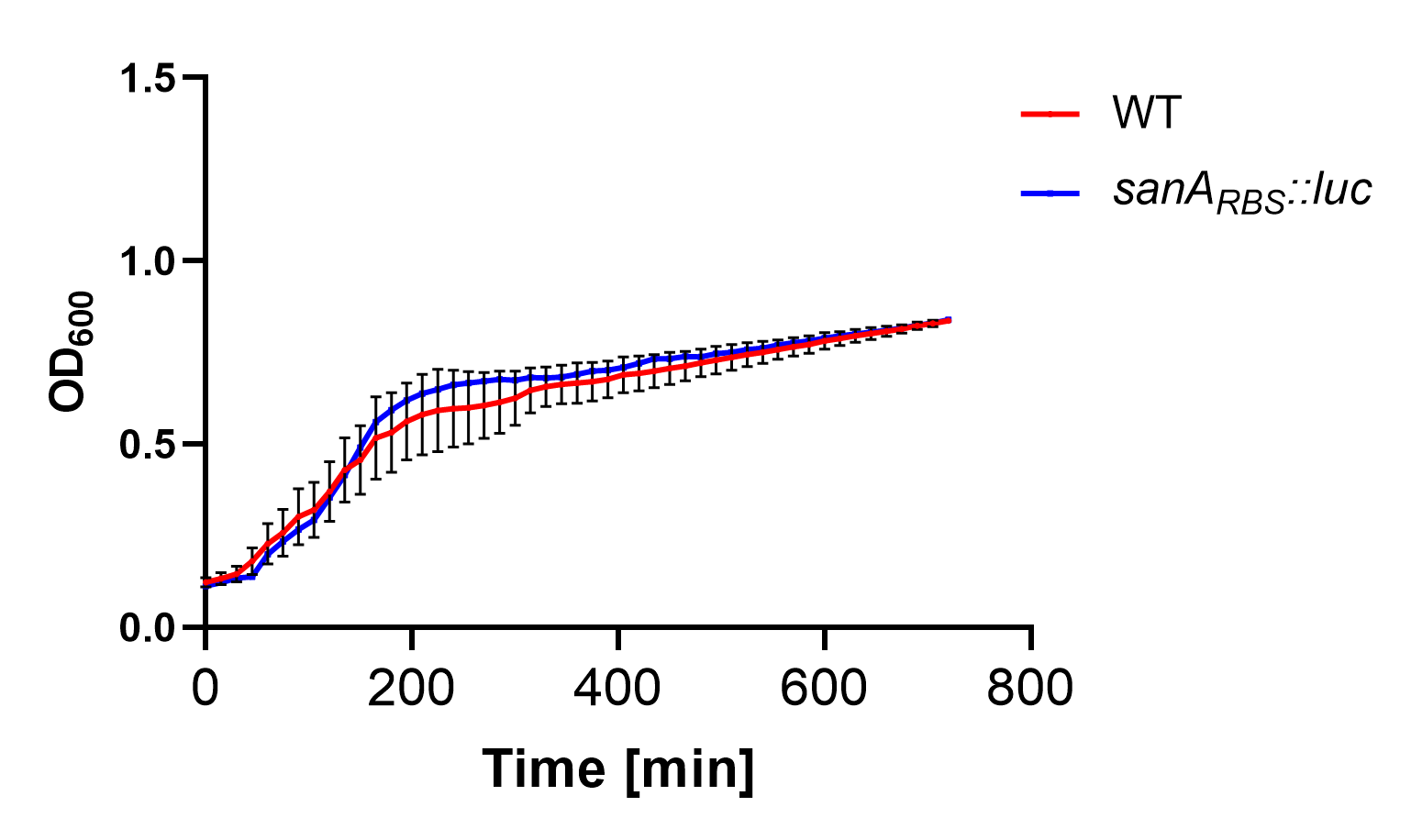

Supplement: Figure S1 — Growth curves of S. Typhimurium 4/74 WT and sanARBS::luc in LB medium. [file spectrum.02833-24-s0002.tif]

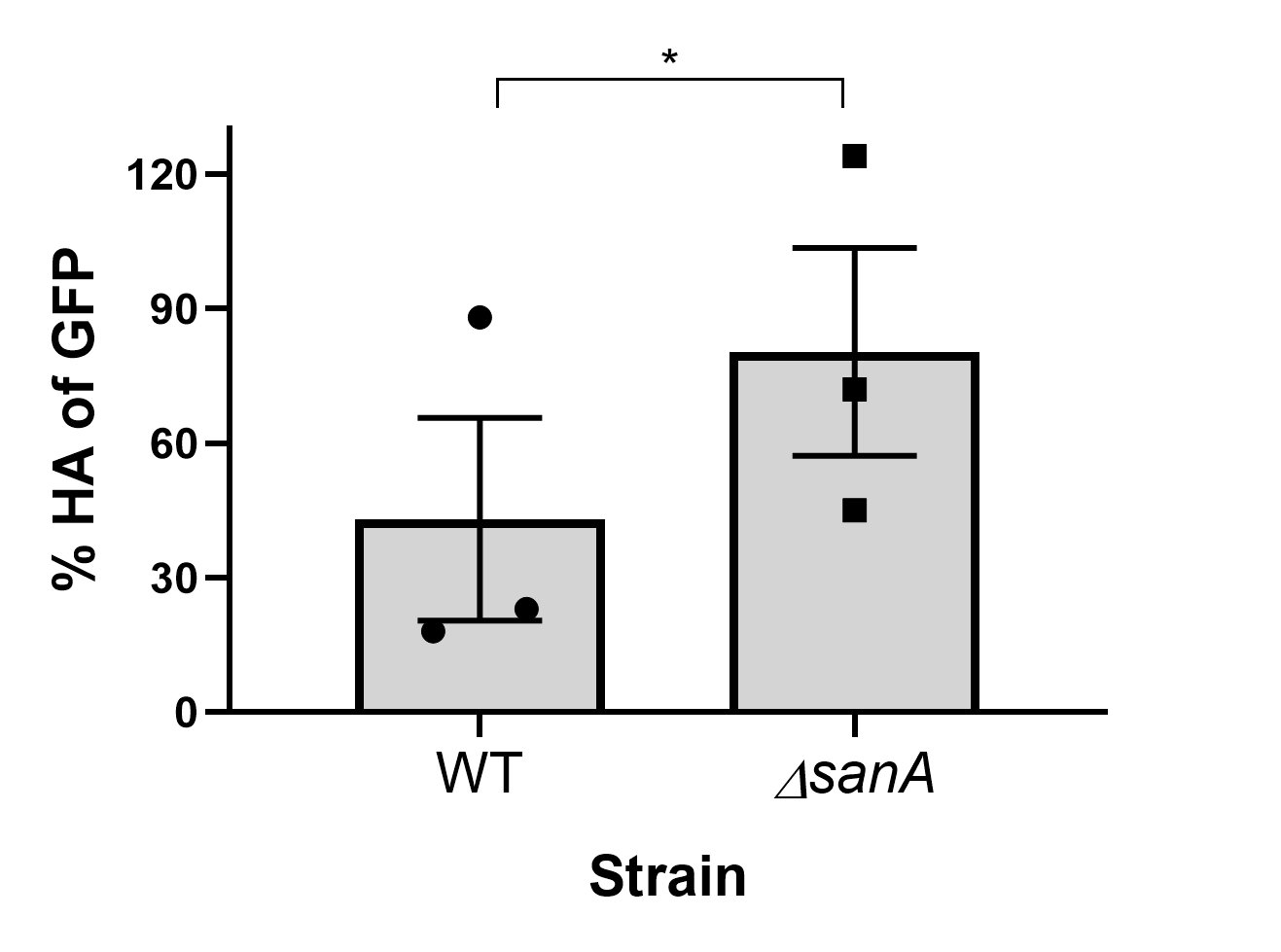

Supplement: Figure S2 — Densitometric analysis of protein bands imaged with the ChemiDoc MP. [file spectrum.02833-24-s0003.tif]

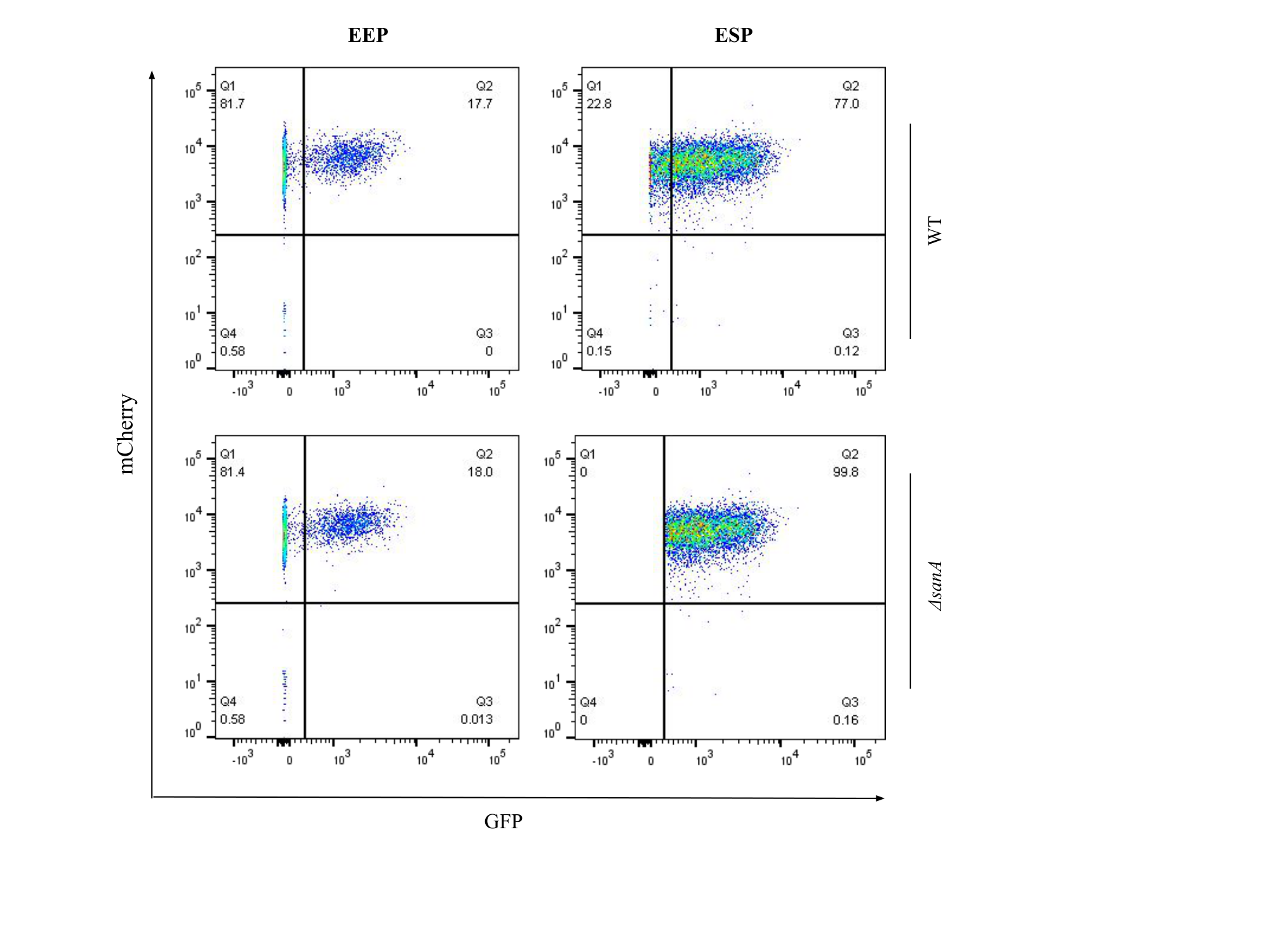

Supplement: Figure S3 — Fraction of cells expressing sicA. Fraction of cells in the ON state was determined relative to the negative control (100% in the OFF state), which consisted of the measured fluorescence of cells not expressing the GFP. EEP: early exponential growth phase corresponding to OD600=0.5; ESP: early stationary growth phase corresponding to OD600=2.0. [file spectrum.02833-24-s0004.tiff]

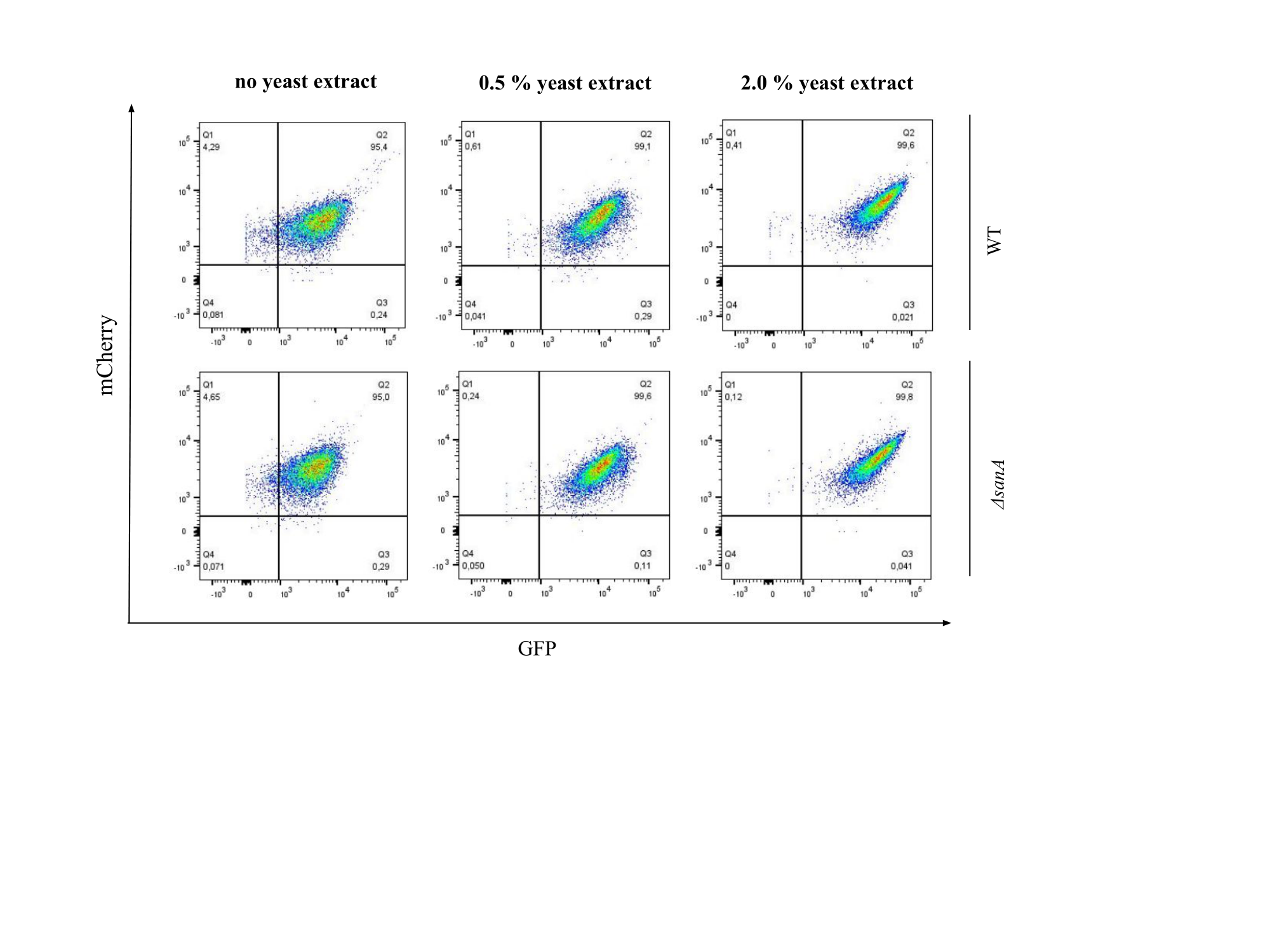

Supplement: Figure S4 — Fraction of cells expressing sicA. Fraction of cells in the ON state was determined relative to the negative control (100% in the OFF state), which consisted of the measured fluorescence of cells not expressing the GFP. [file spectrum.02833-24-s0005.tiff]

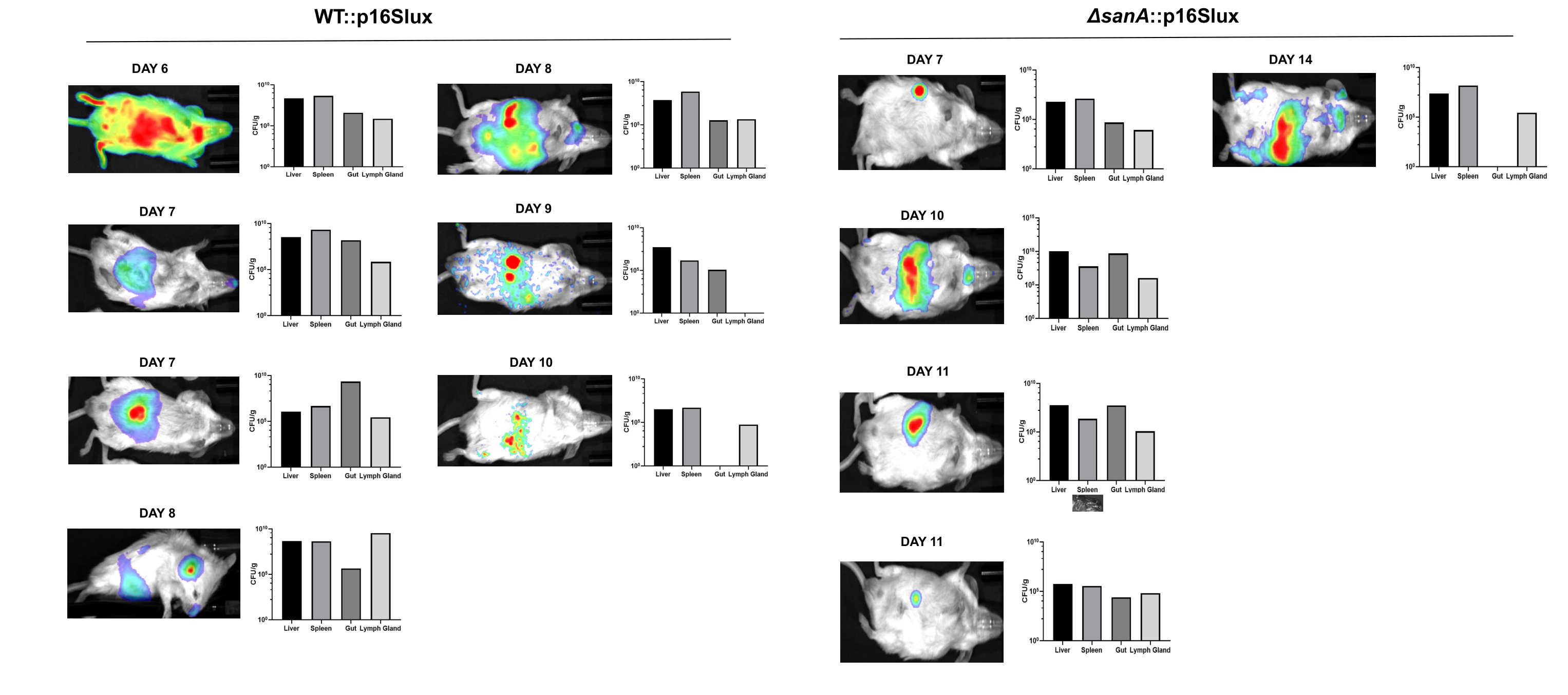

Supplement: Figure S5 — Monitoring of WT::p16Slux S. Typhimurium and ΔsanA::p16Slux infections in mice using whole-body bioluminescence imaging (BLI). [file spectrum.02833-24-s0006.tiff]
